# The value of synthetic MRI in detecting the brain changes and hearing impairment of children with sensorineural hearing loss

**DOI:** 10.3389/fnins.2024.1365141

**Published:** 2024-06-11

**Authors:** Penghua Zhang, Jinze Yang, Yikai Shu, Meiying Cheng, Xin Zhao, Kaiyu Wang, Lin Lu, Qingna Xing, Guangying Niu, Lingsong Meng, Xueyuan Wang, Liang Zhou, Xiaoan Zhang

**Affiliations:** ^1^Third Affiliated Hospital of Zhengzhou University, Zhengzhou, Henan, China; ^2^Henan University of Science and Technology, Luoyang, Henan, China; ^3^MRI Research, GE Healthcare, Beijing, China

**Keywords:** sensorineural hearing loss, white matter, synthetic MRI, magnetic resonance imaging, brain volume

## Abstract

**Introduction:**

Sensorineural hearing loss (SNHL) can arise from a diverse range of congenital and acquired factors. Detecting it early is pivotal for nurturing speech, language, and cognitive development in children with SNHL. In our study, we utilized synthetic magnetic resonance imaging (SyMRI) to assess alterations in both gray and white matter within the brains of children affected by SNHL.

**Methods:**

The study encompassed both children diagnosed with SNHL and a control group of children with normal hearing {1.5-month-olds (*n* = 52) and 3-month-olds (*n* = 78)}. Participants were categorized based on their auditory brainstem response (ABR) threshold, delineated into normal, mild, moderate, and severe subgroups.Clinical parameters were included and assessed the correlation with SNHL. Quantitative analysis of brain morphology was conducted using SyMRI scans, yielding data on brain segmentation and relaxation time.Through both univariate and multivariate analyses, independent factors predictive of SNHL were identified. The efficacy of the prediction model was evaluated using receiver operating characteristic (ROC) curves, with visualization facilitated through the utilization of a nomogram. It's important to note that due to the constraints of our research, we worked with a relatively small sample size.

**Results:**

Neonatal hyperbilirubinemia (NH) and children with inner ear malformation (IEM) were associated with the onset of SNHL both at 1.5 and 3-month groups. At 3-month group, the moderate and severe subgroups exhibited elevated quantitative T1 values in the inferior colliculus (IC), lateral lemniscus (LL), and middle cerebellar peduncle (MCP) compared to the normal group. Additionally, WMV, WMF, MYF, and MYV were significantly reduced relative to the normal group. Additionally, SNHL-children with IEM had high T1 values in IC, and LL and reduced WMV, WMF, MYV and MYF values as compared with SNHL-children without IEM at 3-month group. LL-T1 and WMF were independent risk factors associated with SNHL. Consequently, a prediction model was devised based on LL-T1 and WMF. ROC for training set, validation set and external set were 0.865, 0.806, and 0.736, respectively.

**Conclusion:**

The integration of T1 quantitative values and brain volume segmentation offers a valuable tool for tracking brain development in children affected by SNHL and assessing the progression of the condition's severity.

## Introduction

Congenital sensorineural hearing loss (SNHL) denotes deafness occurring before language development, typically during pregnancy, impacting auditory neural pathways. Approximately 1.2–1.7 cases per 1,000 live births lead to permanent childhood hearing loss due to SNHL (Korver et al., 2010). Delayed diagnosis in infants and young children with SNHL can profoundly hinder learning, affecting language acquisition, memory formation, and cognitive development (Surprenant and Didonato, 2014; Slade et al., 2020; Johnson et al., 2021; Shende and Mudar, 2023).

While the auditory brainstem response (ABR) test is commonly utilized for hearing screening in newborns, more quantitative and sensitive measures are necessary for early and precise diagnosis. Magnetic resonance imaging (MRI) is pivotal in diagnosing and monitoring disease progression and treatment responses (Van Der Weijden et al., 2023). Techniques such as Diffusion Tensor Imaging (DTI), Diffusion Kurtosis Imaging (DKI), and Functional Magnetic Resonance Imaging (fMRI) have been instrumental in diagnosing SNHL and studying brain development in affected infants (Wang et al., 2019). However, conventional MRI methods (T1WI, T2WI) lack the ability for quantitative analysis of brain region changes. Moreover, most studies involve subjects older than 2 years (Wang et al., 2023), potentially limiting the efficacy of interventions aimed at improving language discrimination abilities. While techniques like DTI, DKI, and fMRI offer quantitative analysis, they often necessitate longer scan durations.

Synthetic Magnetic Resonance Imaging (SyMRI) is an innovative technology for relaxation quantification imaging, delivering T1 and T2 relaxation times along with proton density (PD) in a single scan within clinically acceptable acquisition times (Chari and Chan, 2017; Goncalves et al., 2018). This approach offers absolute measurements of tissue microstructure, enhancing the objectivity of disease assessment. Unlike traditional methods, SyMRI allows adjustments of parameters like repetition time, echo time, and inversion time based on mathematical calculations rather than predefined settings (Gulani et al., 2004; Ji et al., 2022). This advancement reduces brain diagnostic study durations to ~5 min with SyMRI, potentially enhancing throughput and minimizing the need for rescans, while delivering valuable quantitative data (Warntjes et al., 2008). SyMRI software, such as Synthetic MR from Linköping, Sweden, streamlines the generation of synthetic quantitative images. It offers fully automated volumetric parameters based on anticipated quantitative values for various brain tissue types (West et al., 2012). Integrated into radiology picture archiving and communication systems, this software enables rapid brain volume analysis in under 1 min (Granberg et al., 2016; Vanderhasselt et al., 2020).

Utilizing SyMRI technology, each voxel within an MRI scan can be categorized into four components: white matter (WM), gray matter (GM), cerebrospinal fluid (CSF), and non-WM/GM/CSF (NON). The measurement of SyMRI volume has been extensively investigated in both pediatric and adult populations. Previous case reports have highlighted the efficacy of SyMRI in diagnosing conditions like Sturge-Weber syndrome (Andica et al., 2016). Moreover, SyMRI enables the synthesis of Gd-enhanced FLAIR images post-acquisition, while Gd-enhanced synthetic Double Inversion Recovery (DIR) can aid in accentuating subtle meningeal enhancements (Andica et al., 2017). SyMRI scans have demonstrated superior plaque detection in multiple sclerosis (MS) compared to conventional MRI (Granberg et al., 2016). Additionally, the utilization of synthetic DIR and Phase-Sensitive Inversion Recovery (PSIR) images may facilitate the identification of intra-cortical or mixed WM-GM lesions (Miller et al., 1998). Studies by Vagberg et al. (2013) have validated SyMRI volumetric analysis as a reliable method for determining brain parenchymal fraction (BPF) in MS, showing that BPF is notably lower in pediatric MS cases, primarily due to GM loss (Yeh et al., 2009). These quantitative values are invaluable in evaluating brain tumors, aiding in differentiation between glioblastomas and metastases (Badve et al., 2017), as well as revealing the internal structure of tumors and lesions in MS (Granberg et al., 2016; Chen et al., 2021; Nunez-Gonzalez et al., 2022). While research on brain relaxation time in SNHL, particularly in children within the first year, is lacking, the potential for SyMRI in exploring this area remains untapped.

In our study, we employed SyMRI to examine the quantitative T1, T2, and PD values across 10 brain regions and 12 brain segmentations in children with SNHL at 1.5 and 3 months of age. Our results offer significant insights for clinical diagnosis and early developmental research in children affected by SNHL.

## Materials and methods

### Participants and clinical assessments

The study received approval from the local ethics committee. A discovery cohort of 80 children diagnosed with SNHL participated and 33 children have normal ABR threshold, in which 52 children tested at 1.5 months and 61 tested at 3 months. An external cohort included 17 children tested at 3 months, comprising nine children diagnosed with SNHL and eight children were normal. All participants underwent ABR testing to determine their hearing thresholds. The severity of hearing loss was categorized as mild (31–50 dB), moderate (51–70 dB), or severe (>70 dB) for each ear.

Inclusion criteria encompassed right-handed children with SNHL identified through hearing screening tests at 1.5 and 3 months post-birth, with bilateral ABR thresholds exceeding 30 dB. Exclusion criteria involved the presence of severe neurological disorders such as epilepsy and congenital leukodystrophy, cognitive impairments like autism and severe hyperactivity syndrome, and a history of treatment for ear-related infections.

### Imaging examinations

SyMRI was conducted on a 3.0 T scanner (SIGNA Pioneer; GE Healthcare, Waukesha, WI, USA) equipped with a 21-channel head coil for all participants. Prior to scanning, children were sedated with midazolam (intramuscular or intravenous administration: 0.05–0.1 mg/kg/time via slow injection for 5 min) and immobilized using a MedVac vacuum device (CFI Medical Solutions, Fenton, Michigan). Ear protection was ensured with neonatal earmuffs covered by headphones. Parental consent was obtained before MRI and sedation. The sequence parameters for SyMRI were set as follows: Field of View (FOV) = 200 mm, slice thickness = 3 mm, slice gap = 0.5 mm, number of slices = 36, TR/TE = 4,230/20.4 ms, NEX=1, with an acquisition time of 5 min and 8 s. Quantification maps (T1, T2, and PD) were generated using the vendor-provided program (SyMRI 8.0; SyntheticMR, Linköping, Sweden).

### Measurements of quantitative values

Following the scans, two neurology specialists meticulously reviewed all scan sequences to eliminate any macroscopic pathology. The SyMRI sequence image guide supplier's program (SyMRI 8.0, Synthetic MR, Linköping, Sweden) was employed to automatically generate T1 and T2 mapping diagrams. The regions of interest (ROIs) for this study were primarily delineated by the co-first author, possessing 7 and 6 years of experience in imaging diagnosis, respectively. All findings underwent thorough review and verification by the corresponding authors and imaging instructors of this study, each with 20 years of imaging diagnosis expertise. For manual operations on T1 and T2 mapping diagrams, the ITK-SNAP 3.8.0 software was utilized. Ten ROIs were sketched, including the semioval center (SC), frontal lobe (FL), posterior limb of the internal capsule (PLIC), genu of the corpus callosum (GCC), splenium of the corpus callosum (SCC), caudate nucleus (CN), globus pallidus (GP), inferior colliculus (IC), lateral lemniscus (LL), and middle cerebellar peduncle (MCP). Each ROI was meticulously placed to ensure precise anatomical positioning, minimizing interference from cerebrospinal fluid and surrounding anatomical structures. T1 and T2 values for each ROI were measured thrice, and their averages were computed. Subsequently, the mean values of symmetrical parts from both brain hemispheres were calculated post-measurement.

### MR volumetric calculations

The raw data obtained from SyMRI underwent further processing with the SyMRI 8.0 post-processing software to derive brain segmentation volume and relaxation values. This included parameters such as white matter volume (WMV), gray matter volume (GMV), cerebrospinal fluid volume (CSF), myelin volume (MYV), brain parenchymal volume (BPV), intracranial volume (ICV), non-WM/GM/CSF (NON), white matter fraction (WMF = WMV/BPV), myelin fraction (MYF = MYV/BPV), gray matter fraction (GMF = GMV/BPV), NONF = NON/BPV, and cerebrospinal fluid fraction (CSFF = CSF/ICV).

### Construction and validation of the prediction model

Parameters including IC-T1, LL-T1, MCP-T1, WMV, WMF, MYV, and MYF were chosen for children examined at 3 months. The discovery cohort of 61 samples was randomly divided into training and validation sets in a ratio of 55–45%, respectively. The external validation set contained 17 samples, including eight normal samples and nine SNHL samples. Univariate analysis was conducted, and variables with *p*-values < 0.05 were included for multivariate analysis using the bidirectional stepwise regression method in training set. A generalized linear model was then employed to build the prediction model. Evaluation of the model's efficacy was performed using a ROC curve, and visualization of a nomogram was facilitated using the R packages “pROC” and “regplot”.

### Statistical analysis

Data analysis was conducted utilizing R software (version 4.0.1). Analysis of variance (ANOVA) was employed to assess differences among variables across the normal, mild, moderate, and severe groups. The Wilcoxon test was utilized for non-normally distributed data to compare differences between two groups, while the Student's *t*-test was applied for normally distributed data. A significance level of *p* < 0.05 was considered statistically significant for all analyses.

## Results

### Correlation of clinical parameters and onset of SNHL

**Figure 1:**
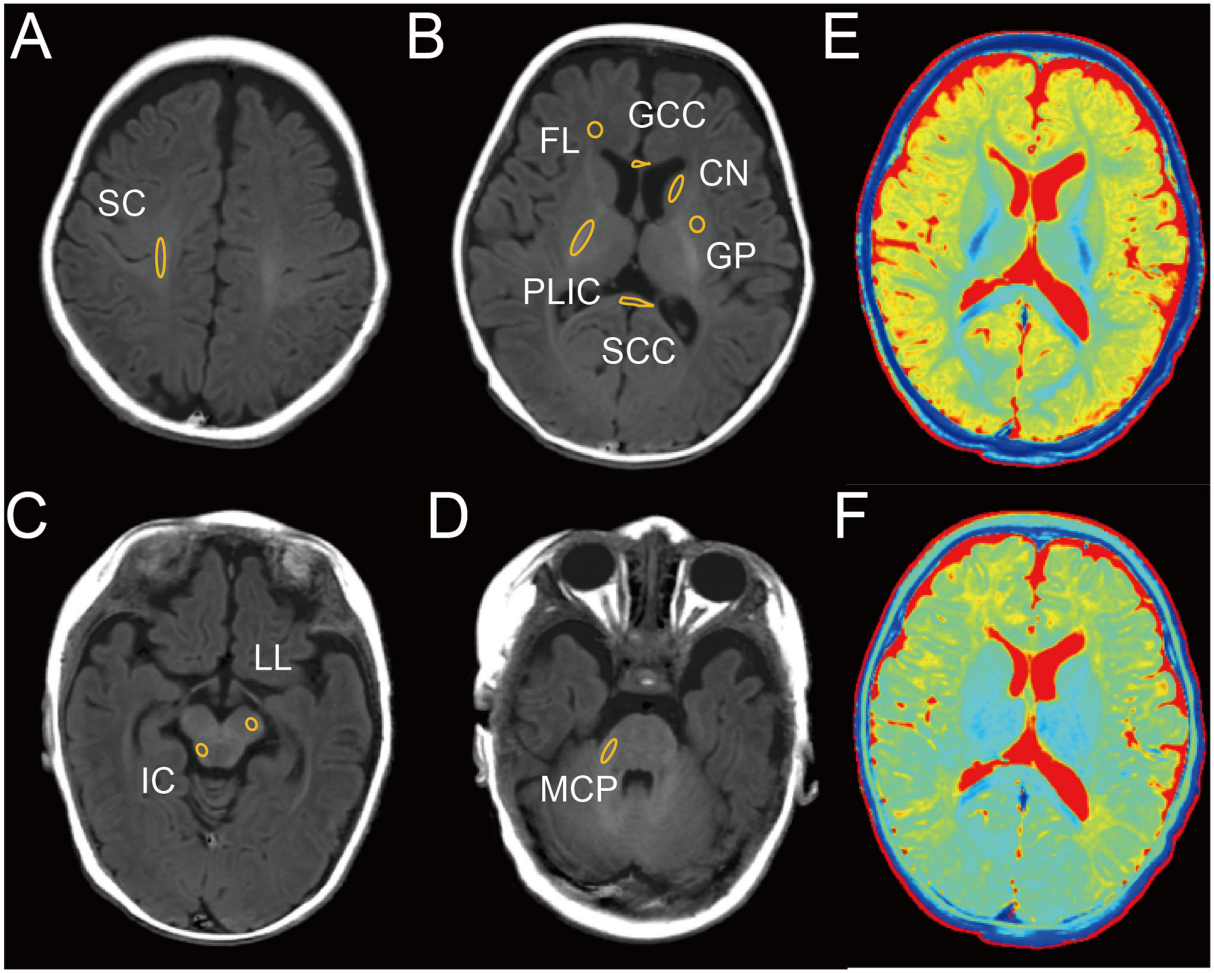
Representative image of male infant brain at 3 months with delineation of ROIs on T1 image. **(A)** Semioval center (SC). **(B)** Frontal lobe (FL), posterior limb of the internal capsule (PLIC), genu of the corpus callosum (GCC), splenium of the corpus callosum (SCC), caudate nucleus (CN), globus pallidus (GP). **(C)** Inferior colliculus (IC), lateral lemniscus (LL). **(D)** Middle cerebellar peduncle (MCP). **(E)** T1 map. **(F)** T2 map.

To assess the diagnostic efficacy of SyMRI for SNHL, we conducted evaluations on a cohort of 52 children at 1.5 months and 61 children at 3 months. The sample was categorized into four groups based on disease severity: normal, mild, moderate, and severe. Tables 1, 2 provide comprehensive clinical details of these children. Notably, no significant differences were detected in age, birth method, birth weight, or sex across the normal, mild, moderate, and severe subgroups. Next, we evaluated the correlation of clinical complications of newborns and pregnant women and onset of SNHL. Results demonstrated that neonatal hyperbilirubinemia (NH) and children with inner ear malformation (IEM) were associated with high incidence of SNHL (Tables 3, 4) both at 1.5 and 3-month group. Next, we used SyMRI to calculate the T1, T2 and PD values as well as automatic whole-brain volume segmentation. Our analysis focused on 10 ROIs, including SC, FL, PLIC, GCC, SCC, CN, GP, IC, LL, and MCP (Figures 1A–D). Additionally, Figures 1E, F depict representative T1 and T2 quantitative maps, respectively.

**Table 1:** Summary of participant characteristics in the 1.5-month group.

	**Normal (*n* =14)**	**Mild (*n* = 16)**	**Moderate (*n* =13)**	**Severe (*n* = 9)**	***p*-value**
Sex (female, male)	6, 8	8, 8	6, 7	3, 6	0.96
Birth method (natural delivery, cesarean section)	8, 6	10, 6	9, 4	5, 4	0.868
Birth weight (g)	3,325 (3,112.5, 3,662.5)	3,050 (2,900, 3,512.5)	3,200 (3,000, 3,800)	3,175 (3,075, 3,487.5)	0.559
Gestational age at birth (weeks)	39.29 (38.25, 40.75)	39.93 (39, 40.21)	39.43 (37.86, 40.43)	39.43 (38.79, 40.29)	0.651
dB hearing loss: left ear	\	43.75 ± 6.191	63.85 ± 6.50	92.22 ± 8.33	<0.001
dB hearing loss: right ear	\	45.63 ± 5.12	64.62 ± 6.60	94.44 ± 5.27	<0.001

**Table 2:** Summary of participant characteristics in the 3-month group.

	**Normal (*n* =19)**	**Mild (*n* = 15)**	**Moderate (*n* =19)**	**Severe (*n* = 8)**	***p*-value**
Sex (female, male)	10, 9	7, 8	11, 8	6, 2	0.655
Birth method (natural delivery, cesarean section)	9, 10	9, 6	9, 10	4, 4	0.871
Birth weight (g)	3,286.84 ± 421.26	3,265.67 ± 542.65	3,323.68 ± 390.29	3,481.25 ± 465.17	0.953
Gestational age at birth (weeks)	39.71 (38.64, 40)	39.57 (38.86, 39.93)	39.29 (38.29, 40)	39.29 (38.79, 40.18)	0.719
dB hearing loss: left ear	\	44.00 ± 6.33	62.11 ± 7.13	87.50 ± 10.35	<0.001
dB hearing loss: right ear	\	45.33 ± 5.16	62.11 ± 7.13	93.75 ± 7.44	<0.001

**Table 3:** Correlation of clinical parameters and SNHL at 1.5-month group.

**Clinical parameters**	**Total (*n* = 52)**	**Normal (*n* = 14)**	**SNHL (*n* = 38)**	***p***
Premature birth, *n* (%)				1
No	36 (69)	10 (71)	26 (68)	
Yes	16 (31)	4 (29)	12 (32)	
NH, *n* (%)				0.03
No	30 (58)	12 (86)	18 (47)	
Yes	22 (42)	2 (14)	20 (53)	
GDM, *n* (%)				0.746
No	34 (65)	10 (71)	24 (63)	
Yes	18 (35)	4 (29)	14 (37)	
HDP, *n* (%)				1
No	42 (81)	11 (79)	31 (82)	
Yes	10 (19)	3 (21)	7 (18)	
CMV infection, *n* (%)				0.729
No	39 (75)	10 (71)	29 (76)	
Yes	13 (25)	4 (29)	9 (24)	
IEM, *n* (%)				0.002
No	36 (69)	14 (100)	22 (58)	
Yes	16 (31)	0 (0)	16 (42)	

NH, neonatal hyperbilirubinemia; HDP, pregnancy-induced hypertension; GDM, gestational diabetes mellitus; CMV, cytomegalovirus; IEM, inner ear malformation.

**Table 4:** Correlation of clinical parameters and SNHL at 3-month group.

**Clinical parameters**	**Total (*n* = 52)**	**Normal (*n* = 14)**	**SNHL (*n* = 38)**	***p***
Premature birth, *n* (%)				0.803
No	42 (69)	14 (74)	28 (67)	
Yes	19 (31)	5 (26)	14 (33)	
NH, *n* (%)				0.022
No	30 (49)	14 (74)	16 (38)	
Yes	31 (51)	5 (26)	26 (62)	
GDM, *n* (%)				0.436
No	39 (64)	14 (74)	25 (60)	
Yes	22 (36)	5 (26)	17 (40)	
HDP, *n* (%)				1
No	47 (77)	15 (79)	32 (76)	
Yes	14 (23)	4 (21)	10 (24)	
CMV infection, *n* (%)				0.707
No	52 (85)	17 (89)	35 (83)	
Yes	9 (15)	2 (11)	7 (17)	
IEM, *n* (%)				0.003
No	47 (77)	19 (100)	28 (67)	
Yes	14 (23)	0 (0)	14 (33)	

NH, neonatal hyperbilirubinemia; HDP, pregnancy-induced hypertension; GDM, gestational diabetes mellitus; CMV, cytomegalovirus; IEM, inner ear malformation.

### Measurements of quantitative parameters correlated with SNHL

**Figure 2:**
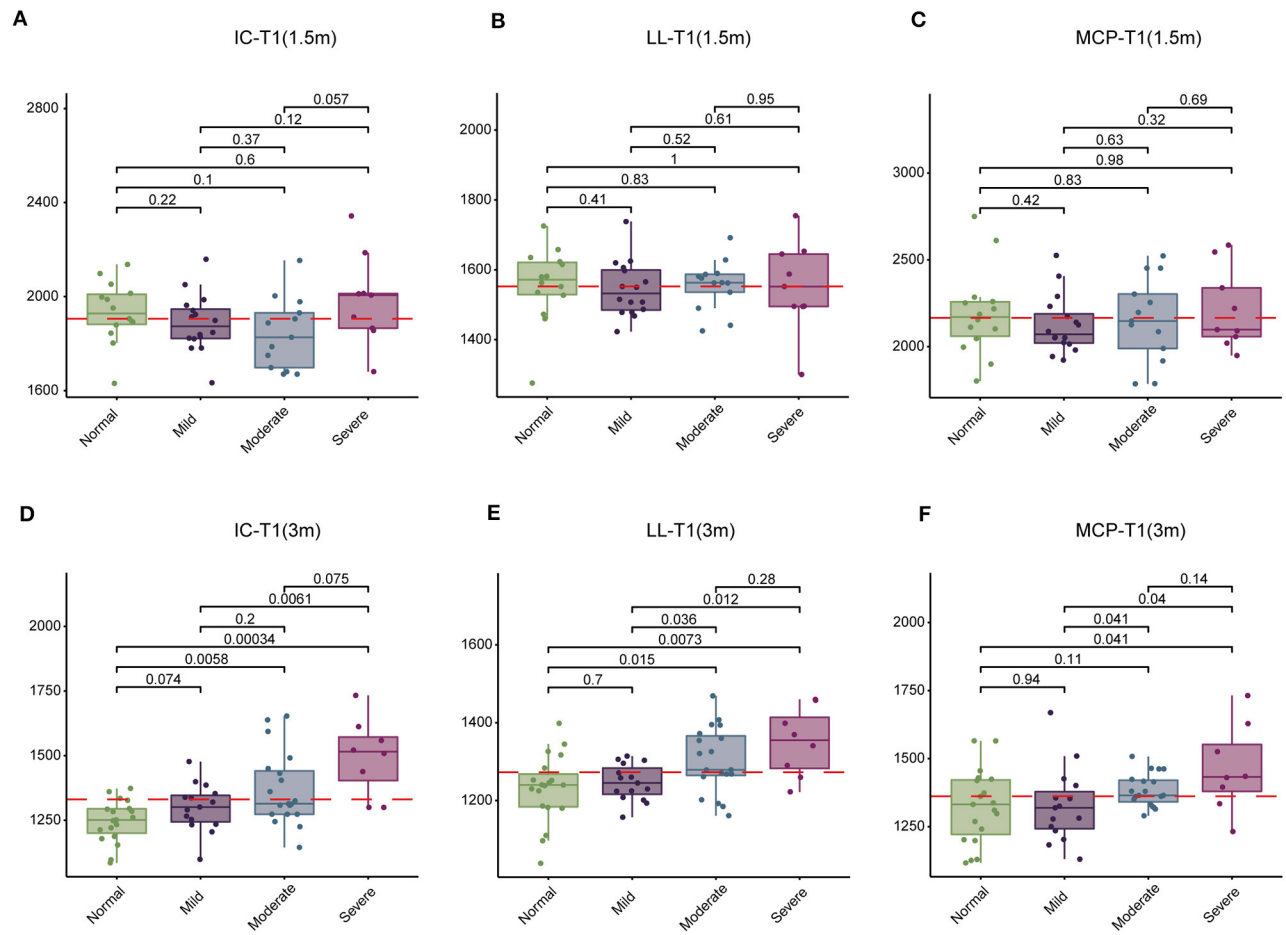
Comparison of T1 values in the inferior colliculus (IC), lateral lemniscus (LL), and middle cerebellar peduncle (MCP) among four groups of children at 1.5 and 3 months. **(A–C)** Comparison of T1 values among normal, mild, moderate, and severe subgroups at 1.5 months. **(D–F)** Comparison of T1 values among normal, mild, moderate, and severe subgroups at 3 months. T1 values are presented in milliseconds.

Initially, we conducted an analysis of T1, T2, and PD values in the brains of children tested at 1.5- and 3-month groups across normal, mild, moderate, and severe subgroups. At 1.5-month group, no significant changes were observed in T1, T2, and PD values across the four subgroups (Table 5). However, at 3-month group, significant differences were noted in T1 values within the IC, LL, and MCP regions across the four subgroups, while T2 and PD values remained relatively stable (Table 6). Subsequent pairwise comparisons of T1 values within IC, LL, and MCP between the groups at 1.5 and 3 months revealed no significant differences at 1.5 month-group (Figures 2A–C). However, at 3 month-group, while no significant change in T1 values was observed between the normal and mild subgroups in IC, LL, and MCP, there was a notable progressive increase in T1 values from moderate to severe subgroups compared to the normal subgroup in IC and LL, with a similar trend observed in MCP, albeit only significantly in the severe group (Figures 2D–F). These findings underscore the potential of T1 values to serve as a more sensitive indicator of SNHL progression by 3 months.

**Table 5:** T1, T2, and PD values in different regions of the brain in the 1.5-month group.

**Variables**	**Total (*n* = 52)**	**Normal (*n* = 14)**	**Mild (*n* = 16)**	**Moderate (*n* = 13)**	**Severe (*n* = 9)**	***p*-value**
SCC-T1	2,217 (2,159, 2,343.25)	2,206 (2,163, 2,310)	2,182 (2,136.75, 2,368.25)	2,203 (2,164, 2,338)	2,294 (2,218, 2,295)	0.487
SCC-T2	278.56 ± 16.04	278.71 ± 14.52	285.06 ± 14.88	269.23 ± 17.21	280.22 ± 14.47	0.063
SCC-PD	108.26 ± 2.83	108.43 ± 2.93	106.99 ± 2.77	108.97 ± 3.02	109.23 ± 1.99	0.16
GCC-T1	2,865 (2,749, 2,912)	2,867.5 (2,814, 2,902)	2,793.5 (2,662, 2,873.25)	2,877 (2,783, 2,912)	2,832 (2,774, 3,000)	0.397
GCC-T2	211 (204, 227)	214 (204, 230.75)	211.5 (205, 229.75)	205 (204, 221)	208 (203, 211)	0.517
GCC-PD	128.85 (127.07, 130.57)	128.05 (124.82, 129)	128.5 (127.6, 129.82)	129.7 (129, 131.3)	128.7 (128.2, 131)	0.271
IC-T1	1,905.79 ± 149.91	1,935 ± 130.07	1,887 ± 122.25	1,841.69 ± 150.92	1,986.33 ± 193.43	0.121
IC-T2	163.35 ± 9.84	167.21 ± 7.94	163.62 ± 10.61	159.92 ± 10.13	161.78 ± 10.1	0.269
IC-PD	115.73 ± 4.27	115.65 ± 5.36	116.18 ± 4.64	114.97 ± 3.92	116.14 ± 2.2	0.885
LL-T1	1,552.56 ± 93.02	1,557.36 ± 107.49	1,544.88 ± 78.77	1,556.38 ± 71.93	1,553.22 ± 129.65	0.984
LL-T2	158.19 ± 8.67	158.71 ± 8.19	160.44 ± 7.96	155.46 ± 9.84	157.33 ± 9.07	0.486
LL-PD	161.97 ± 7.46	161.84 ± 8.24	159.89 ± 6.68	164.18 ± 7.09	162.66 ± 8.28	0.495
PLIC-T1	2,437 (2,343.5, 2,571.5)	2,546 (2,404.5, 2,698.5)	2,397.5 (2,295, 2,494.5)	2,404 (2,349, 2,472)	2,523 (2,364, 2,614)	0.252
PLIC-T2	179.6 ± 12.17	183 ± 14.1	177.44 ± 10.34	179.31 ± 13.24	178.56 ± 11.26	0.656
PLIC-PD	108.89 ± 4.71	109.9 ± 5.51	106.81 ± 3.82	110.03 ± 4.86	109.37 ± 4.04	0.203
FL-T1	3,687.5 (3,524.75, 3,868)	3,777.5 (3,587.5, 3,909.5)	3,630 (3,377.75, 3,810.75)	3,646 (3,535, 3,836)	3,696 (3,529, 4,313)	0.376
FL-T2	282 (258.5, 316.75)	301.5 (275.25, 326)	279 (255.25, 313.75)	282 (259, 316)	276 (257, 343)	0.628
FL-PD	135.7 (134, 136.6)	136.5 (135.45, 137.2)	135.1 (133.18, 136.27)	135.6 (133.4, 136.5)	136.5 (135.6, 136.8)	0.183
CN-T1	2,417.85 ± 164.97	2,476.14 ± 162.35	2,354.88 ± 170.53	2,403.62 ± 129.14	2,459.67 ± 186.98	0.191
CN-T2	194.27 ± 16.56	202.14 ± 18.81	188.62 ± 13.76	190.54 ± 15.92	197.44 ± 15.21	0.108
CN-PD	140.15 (137.85, 142.12)	141.05 (139.22, 141.9)	139.65 (136.8, 141.9)	141.4 (139, 143.1)	140.2 (139.4, 141.1)	0.584
GP-T1	1,885.69 ± 107.65	1,907.64 ± 95.52	1,837.69 ± 113.88	1,875.77 ± 90.19	1,951.22 ± 109.95	0.06
GP-T2	229 ± 21.93	239.14 ± 17.16	217.94 ± 24.54	230.46 ± 24.02	230.78 ± 12.66	0.061
GP-PD	119.05 ± 2.16	118.86 ± 1.13	118.83 ± 1.68	119.94 ± 2.46	118.46 ± 3.38	0.376
SC–T1	2,279 (2,044.75, 2,499.5)	2,344 (2,164.5, 2,551.25)	2,236.5 (1,992, 2,525.5)	2,207 (1,979, 2,269)	2,421 (2,048, 2,632)	0.064
SC-T2	232.17 ± 28.67	243.43 ± 27.34	228.62 ± 24.44	227.31 ± 22.03	228 ± 43.28	0.406
SC-PD	103.53 ± 4.19	104.96 ± 3.92	103.21 ± 4.3	101.73 ± 3.58	104.44 ± 4.8	0.209
MCP-T1	2,165.9 ± 217.62	2,191.21 ± 250.66	2,126.38 ± 166.23	2,155.62 ± 242.58	2,211.67 ± 230.11	0.777
MCP-T2	173 (160, 189.25)	182.5 (165.25, 190.75)	164 (159.75, 185.25)	176 (162, 180)	164 (159, 184)	0.482
MCP-PD	101.66 ± 6.07	104.62 ± 3.94	100.66 ± 6.37	100.36 ± 6.76	100.72 ± 6.62	0.207

SCC, splenium of the corpus callosum; GCC, genu of the corpus callosum; IC, inferior colliculus; LL, lateral lemniscus; PLIC, posterior limb of the internal capsule; FL, frontal lobe; CN, caudate nucleus; GP, globus pallidus; SC, semioval center; MCP, middle cerebellar peduncle.

**Table 6:** T1, T2, and PD values in different regions of the brain in the 3-month group.

**Variables**	**Total (*n* = 61)**	**Normal (*n* = 19)**	**Mild (*n* = 15)**	**Moderate (*n* = 19)**	**Severe (*n* = 8)**	***p*-value**
SCC-T1	1,739 (1,673, 1,860)	1,704(1,667.5, 1,794.5)	1,702 (1,664, 1,881)	1,747 (1,683, 1,851)	1,780.5 (1,715, 1,828)	0.608
SCC-T2	171.82 ± 14.31	165.37 ± 10.72	176.33 ± 11	173.37 ± 18.4	175 ± 13.08	0.11
SCC-PD	83.9 (83, 85.9)	83.8 (83.3, 86.3)	83 (82.3, 84.5)	85.2 (83.4, 86.8)	84.1 (83.6, 84.78)	0.173
GCC-T1	1,639 (1,596, 1,662)	1,626 (1,599, 1,653)	1,638 (1,553, 1,662.5)	1,647 (1,613, 1,677)	1,634.5 (1,599.75, 1,654.5)	0.666
GCC-T2	145 (142, 155)	145 (142, 157)	147 (143, 158)	142 (137, 155)	143.5 (142.75, 148)	0.366
GCC-PD	82.8 (81.2, 83.7)	82.7 (80.9, 83.45)	82.8 (82, 83.6)	83 (82.1, 83.95)	82.85 (81.2, 83.42)	0.903
IC-T1	1,301 (1,248, 1,386)	1,251 (1,200, 1,294)	1,301 (1,244, 1,346)	1,314 (1,273, 1,441)	1,515.5 (1,403.5, 1,572.25)	<0.001
IC-T2	119 (116, 124)	119 (117, 121)	119 (115, 120)	121 (116.5, 126)	118.5 (112.75, 130.25)	0.887
IC-PD	80.05 ± 3.08	81.02 ± 3.05	80.99 ± 3.07	78.88 ± 2.99	78.72 ± 2.37	0.053
LL-T1	1,272.8 ± 87.74	1,230.89 ± 87.53	1,246.07 ± 46.54	1,303.37 ± 84.85	1,349.88 ± 88.39	0.001
LL-T2	118.33 ± 7.53	117.68 ± 7.52	120.33 ± 5.95	119.05 ± 9.46	114.38 ± 3.29	0.316
LL-PD	81.08 ± 3.31	81.14 ± 3.35	79.73 ± 3.29	82.01 ± 3.35	81.28 ± 2.87	0.265
PLIC -T1	950 (900, 1,024)	966 (928, 1,012.5)	965 (904, 1,005.5)	969 (918, 1,058.5)	891 (885, 900)	0.137
PLIC -T2	106.28 ± 9.05	108.42 ± 8.08	107.87 ± 7	105.64 ± 11.51	99.75 ± 5.37	0.12
PLIC -PD	71 (69, 73.2)	72.2 (69.35, 74.35)	70.3 (69.1, 72.95)	71.4 (69.4, 72.65)	68.85 (68.4, 69.55)	0.286
FL-T1	1,886 (1,772, 1,997)	1,924 (1,837, 2,008.5)	1,886 (1,723.5, 1,958)	1,887 (1,803.5, 2,012)	1,776 (1,760, 1,830.5)	0.126
FL-T2	185 (172, 207)	196 (178.5, 215)	187 (171.5, 205)	183 (179, 207)	169 (164.5, 176)	0.085
FL-PD	86.7 (85.9, 87.3)	87 (85.95, 87.35)	86.5 (85.9, 87.35)	86.5 (85.3, 87.2)	86.8 (86.62, 87.62)	0.695
CN-T1	1,523.51 ± 112.33	1,548.37 ± 100.55	1,506.93 ± 125.6	1,544.53 ± 119.27	1,445.62 ± 60.33	0.121
CN-T2	139 (128, 148)	145 (129.5, 150)	133 (130, 143.5)	139 (127, 149.5)	129 (125, 134.25)	0.372
CN-PD	83.1 (81.3, 84.1)	83.3 (82.7, 83.9)	82.5 (81, 84.3)	81.6 (81.15, 84.35)	82.2 (81.6, 83.3)	0.463
GP-T1	1,453 (1,397, 1,520)	1,450 (1,393, 1,514.5)	1,450 (1,393, 1,524)	1,502 (1,406, 1,564.5)	1,438.5 (1,413.75, 1,453.25)	0.335
GP-T2	134.48 ± 13.67	136.95 ± 11.12	128.93 ± 14.46	138.74 ± 15.03	128.88 ± 10.8	0.096
GP-PD	84.09 ± 1.57	83.85 ± 1.17	84.21 ± 1.3	84.35 ± 2.06	83.81 ± 1.67	0.735
SC–T1	1,435 (1,307, 1,643)	1,435 (1,330, 1,540)	1,456 (1,307, 1,665.5)	1,405 (1,272, 1,567.5)	1,452.5 (1,252.5, 1,829.5)	0.975
SC-T2	151 (138, 163)	150 (144, 154.5)	150 (140.5, 162.5)	153 (131.5, 168.5)	163 (150.5, 170.25)	0.632
SC-PD	81.86 ± 3.48	82.32 ± 3.04	82.01 ± 3.4	81.52 ± 4.28	81.3 ± 2.89	0.868
MCP-T1	1,361.95 ± 127.27	1,323.79 ± 135.16	1,327.93 ± 136.38	1,383.95 ± 59.91	1,464.12 ± 160.52	0.032
MCP-T2	121 (112, 134)	124 (111.5, 135.5)	116 (111, 132)	124 (115, 134.5)	113.5 (110.25, 114)	0.252
MCP-D	79.05 ± 4.28	79.77 ± 4.03	78.89 ± 5.29	78.65 ± 4.18	78.61 ± 3.49	0.854

SCC, splenium of the corpus callosum; GCC, genu of the corpus callosum; IC, inferior colliculus; LL, lateral lemniscus; PLIC, posterior limb of the internal capsule; FL, frontal lobe; CN, caudate nucleus; GP, globus pallidus; SC, semioval center; MCP, middle cerebellar peduncle.

### Detection of brain volume segmentation correlated with SNHL

**Figure 3:**
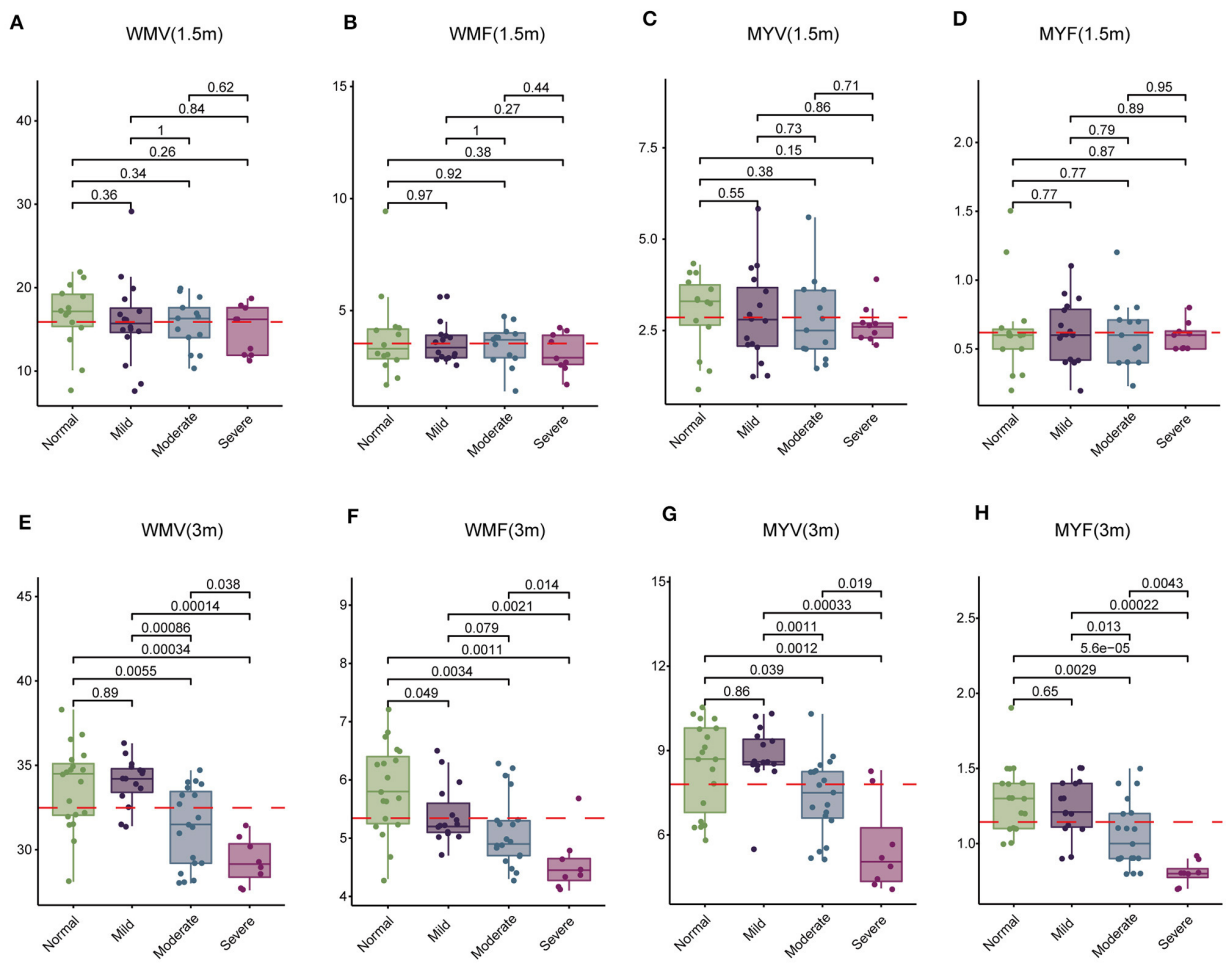
Comparison of white matter volume (WMV), white matter fraction (WMF), myelin volume (MYV), and myelin fraction (MYF) among normal, mild, moderate, and severe subgroups at 1.5 and 3 months. **(A–D)** Comparison at 1.5 months. **(E–H)** Comparison at 3 months.

Subsequently, we examined 12 brain segmentation parameters, comprising WMV, GMV, CSF, BPV, ICV, MYV, NON, WMF, GMF, CSFF, NONF, and MYF, across the normal, mild, moderate, and severe subgroups at 1.5 and 3 months. In line with the T1, T2, and PD findings, at 1.5 month-group, these parameters exhibited no significant differences among the four subgroups (Table 7). However, at 3 months, WMV, WMF, MYV, and MYF displayed distinctions among the four subgroups (Table 8). Subsequent pairwise comparisons of these parameters between each pair of groups at 1.5 and 3 months revealed that in line with above findings, at 1.5-month group, there were no significant difference across these subgroups (Figures 3A–D). At 3 -month group, WMV, MYV, and MYF demonstrated no variance between the normal and mild subgroups, whereas WMF decreased in the mild subgroup. Additionally, at 3 months, WMV, WMF, MYV, and MYF decreased in the moderate and severe subgroups compared to the normal subgroup (Figures 3E–H).

**Table 7:** Summary of brain segmentation in the 1.5-month group.

**Variables**	**Total (*n* = 52)**	**Normal (*n* = 14)**	**Mild (*n* = 16)**	**Moderate (*n* = 13)**	**Severe (*n* = 9)**	***p*-value**
WMV	15.89 ± 3.95	16.63 ± 4.01	16 ± 5.08	15.62 ± 3.07	14.92 ± 2.97	0.788
GMV	507.65 (474.32, 540.88)	497.8 (481.33, 512.25)	507.65 (473.72, 521.68)	502.9 (444.3, 536.2)	548.8 (530.9, 558)	0.269
CSF	61.15 (55.88, 67.38)	61.25 (56.47, 63.08)	60.7 (56.7, 65.2)	61.2 (55.8, 68.5)	66.2 (52, 72.6)	0.986
NON	2.45 (2.08, 3)	2.6 (2.4, 2.88)	2.15 (1.8, 3.52)	2.2 (2.1, 3.3)	2.5 (2.1, 2.8)	0.62
MYV	2.86 ± 1.06	3.03 ± 1.06	2.88 ± 1.24	2.77 ± 1.16	2.68 ± 0.54	0.875
WMF	3.5 (2.8, 4.03)	3.3 (2.85, 4.18)	3.35 (2.9, 3.9)	3.7 (2.9, 4)	2.9 (2.6, 3.9)	0.734
GMF	95.5 (92.38, 96.8)	95.5 (94.15, 96.65)	95.5 (92.25, 96.8)	96.1 (92.4, 96.9)	95.2 (91.6, 96.8)	0.965
CSFF	10.6 ± 2.56	10.89 ± 1.52	10.69 ± 3.32	10.55 ± 2.78	10.03 ± 2.27	0.893
NONF	0.6 (0.5, 0.69)	0.6 (0.5, 0.69)	0.62 (0.55, 0.69)	0.6 (0.58, 0.61)	0.6 (0.5, 0.7)	0.939
MYF	0.6 (0.5, 0.7)	0.6 (0.5, 0.64)	0.6 (0.42, 0.79)	0.6 (0.4, 0.71)	0.6 (0.5, 0.63)	0.982

WMV, volume of white matter; GMV, volume of gray matter; CSF, cerebrospinal fluid; NON, non-WM/GM/CSF; MYV, myelination volume; WMF, white matter fraction; GMF, gray matter fraction; CSFF, cerebrospinal fluid fraction; NONF, NON fraction; MYF, myelin fraction.

**Table 8:** Summary of brain segmentation in the 3-month group.

**Variables**	**Total (*n* = 61)**	**Normal (*n* = 19)**	**Mild (*n* = 15)**	**Moderate (*n* = 19)**	**Severe (*n* = 8)**	***p*-value**
WMV	32.9 (30.8, 34.6)	34.5 (32.05, 35.1)	34.2 (33.4, 34.8)	31.5 (29.2, 33.45)	29.15 (28.38, 30.35)	<0.001
GMV	607.26 ± 29.72	607.94 ± 44.2	607.96 ± 16.14	606.55 ± 24.85	606.01 ± 20.87	0.998
CSF	124.61 ± 22.52	114.48 ± 20.29	124.79 ± 14.03	128.93 ± 25.89	138.07 ± 25.14	0.056
NON	4.79 ± 1.05	4.85 ± 1.48	4.82 ± 0.65	4.81 ± 0.85	4.54 ± 0.98	0.912
MYV	8.3 (6.5, 8.9)	8.7 (6.8, 9.8)	8.6 (8.5, 9.4)	7.5 (6.6, 8.25)	5.05 (4.35, 6.25)	<0.001
WMF	5.2 (4.8, 5.9)	5.8 (5.25, 6.4)	5.2 (5.1, 5.6)	4.9 (4.7, 5.3)	4.45 (4.27, 4.65)	<0.001
GMF	93.08 ± 1.55	93.26 ± 1.78	93.07 ± 1.33	92.92 ± 1.5	93.03 ± 1.71	0.929
CSFF	13 (9.9, 14.2)	12.8 (10.5, 14)	12.1 (9.9, 14.3)	13.2 (9.55, 14.15)	12.2 (8.43, 14.93)	0.992
NONF	0.72 (0.65, 0.82)	0.8 (0.61, 1)	0.72 (0.7, 0.8)	0.76 (0.68, 0.84)	0.73 (0.68, 0.8)	0.889
MYF	1.1 (0.9, 1.4)	1.3 (1.1, 1.4)	1.21 (1.11, 1.4)	1 (0.9, 1.2)	0.8 (0.78, 0.83)	<0.001

WMV, volume of white matter; GMV, volume of gray matter; CSF, cerebrospinal fluid; NON, non-WM/GM/CSF; MYV, myelination volume; WMF, white matter fraction; GMF, gray matter fraction; CSFF, cerebrospinal fluid fraction; NONF, NON fraction; MYF, myelin fraction.

### Correlation of inner ear malformations and SyMRI parameters

**Figure 4:**
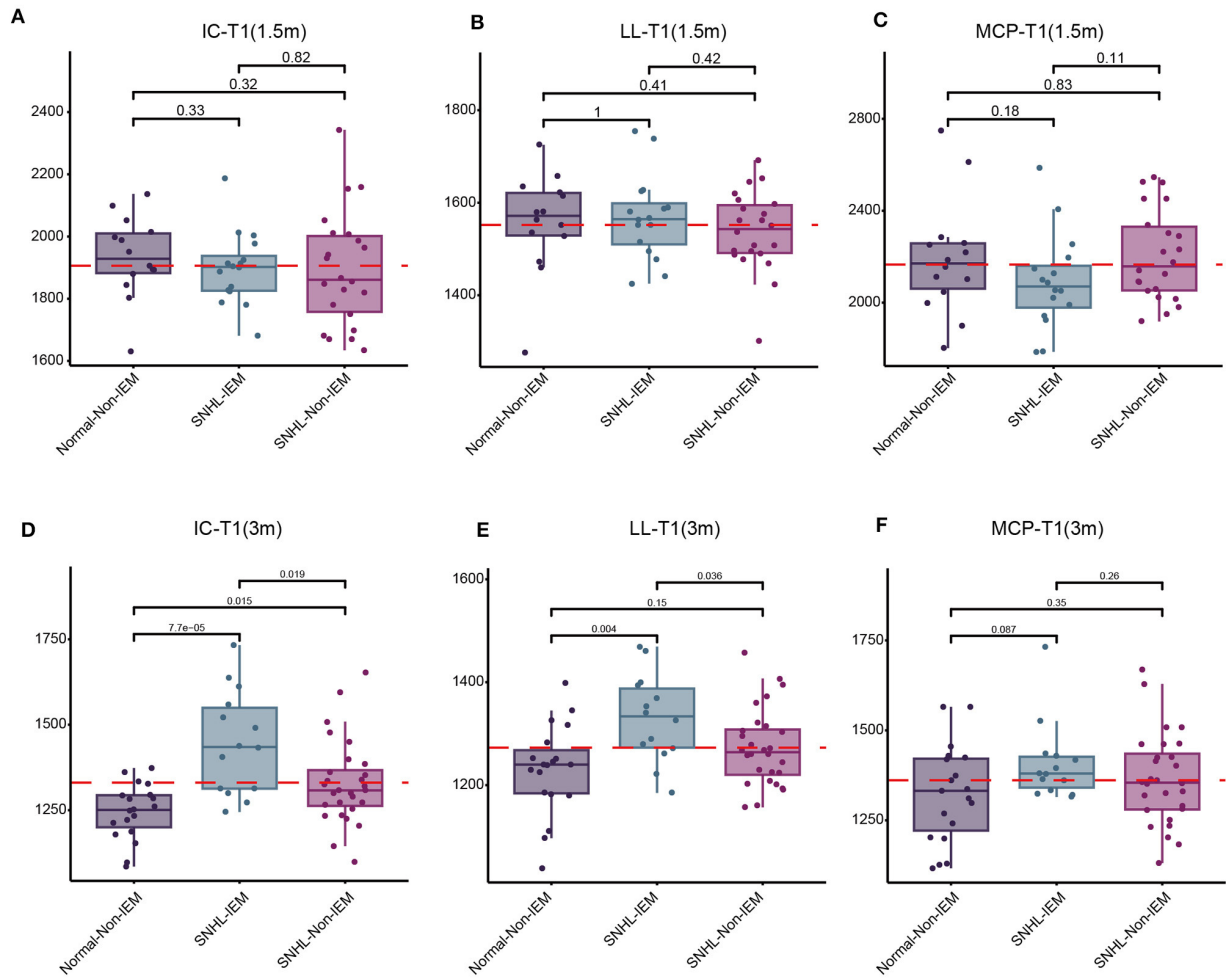
Comparison of T1 values in the inferior colliculus (IC), lateral lemniscus (LL), and middle cerebellar peduncle (MCP) among three groups of children at 1.5 and 3 months. **(A–C)** Comparison of T1 values among Normal-Non-IEM, SNHL-IEM, and SNHL-Non-IEM, subgroups at 1.5 months. **(D–F)** Comparison of T1 values among Normal-Non-IEM, SNHL-IEM, and SNHL-Non-IEM, subgroups at 3 months. T1 values are presented in milliseconds.

**Figure 5:**
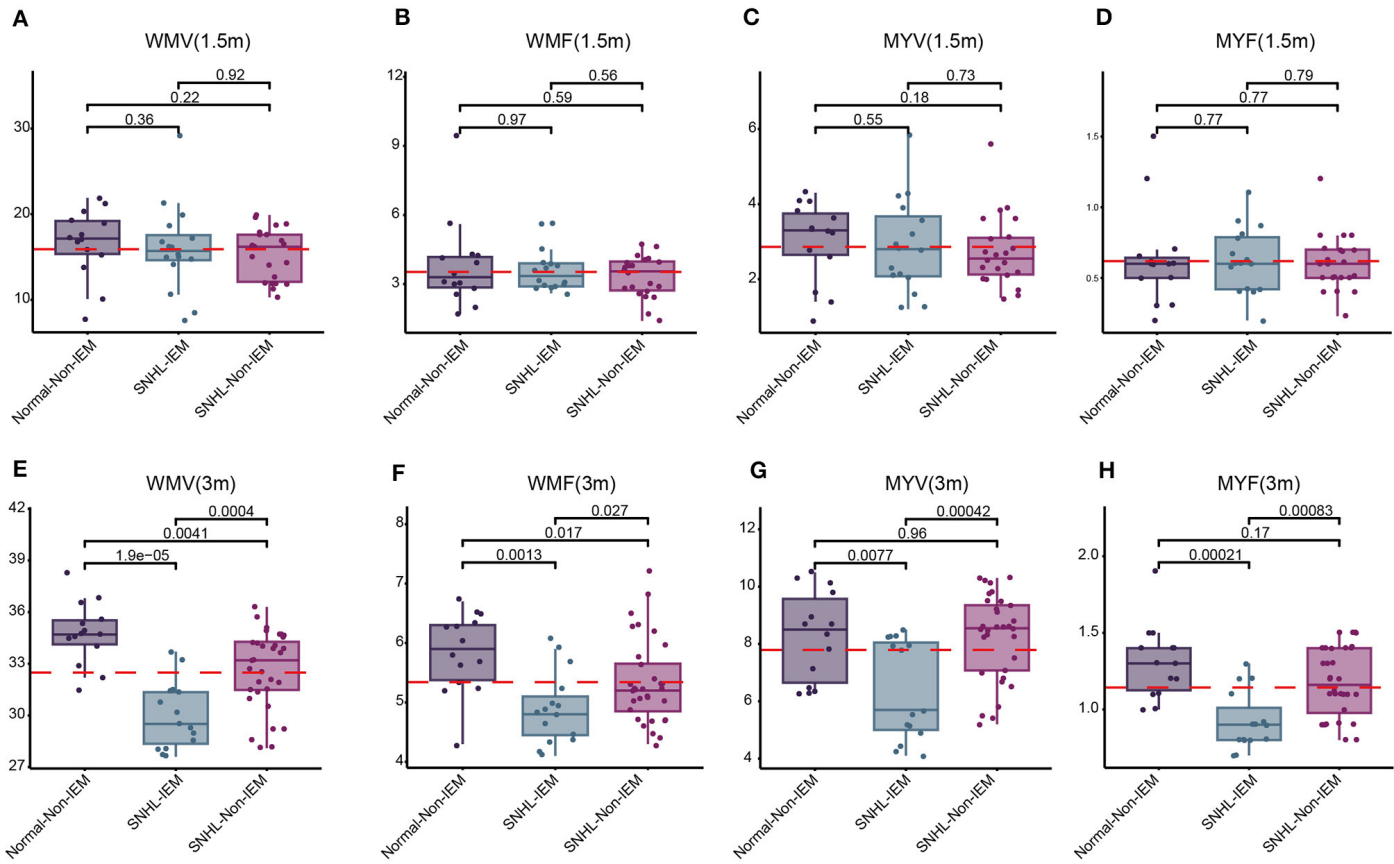
Comparison of white matter volume (WMV), white matter fraction (WMF), myelin volume (MYV), and myelin fraction (MYF) among Normal-Non-IEM, SNHL-IEM, and SNHL-Non-IEM, subgroups at 1.5 and 3 months. **(A–D)** Comparison at 1.5 months. **(E–H)** Comparison at 3 months.

Above findings we found NH and children with IEM were associated with SNHL. Next, we explored the correlation of these two risk factors with SyMRI parameters, including IC-T1, LL-T1, MCP-T1, WMV, WMF, MYV, and MYF. Children were subgrouped according to whether they have this etiology, denoted as Normal-NH, Normal-Non-NH (Non-IEM), SNHL-NH (IEM), and SNHL-Non-NH (Non-IEM). Results demonstrated that both at 1.5 and 3-month groups, there was no significant difference in these parameters between Normal-NH and Normal-Non-NH (Supplementary Figures 1, 2). Instead, we found that SNHL-IEM showed high T1 values in IC and LL, while had low values of WMV, WMF, MYV, and MYF at 3 months, as compared with SNHL-Non-IEM, although there was no difference at 1.5-month group (Figures 4, 5).

### Construction and validation of the prediction model

**Figure 6:**
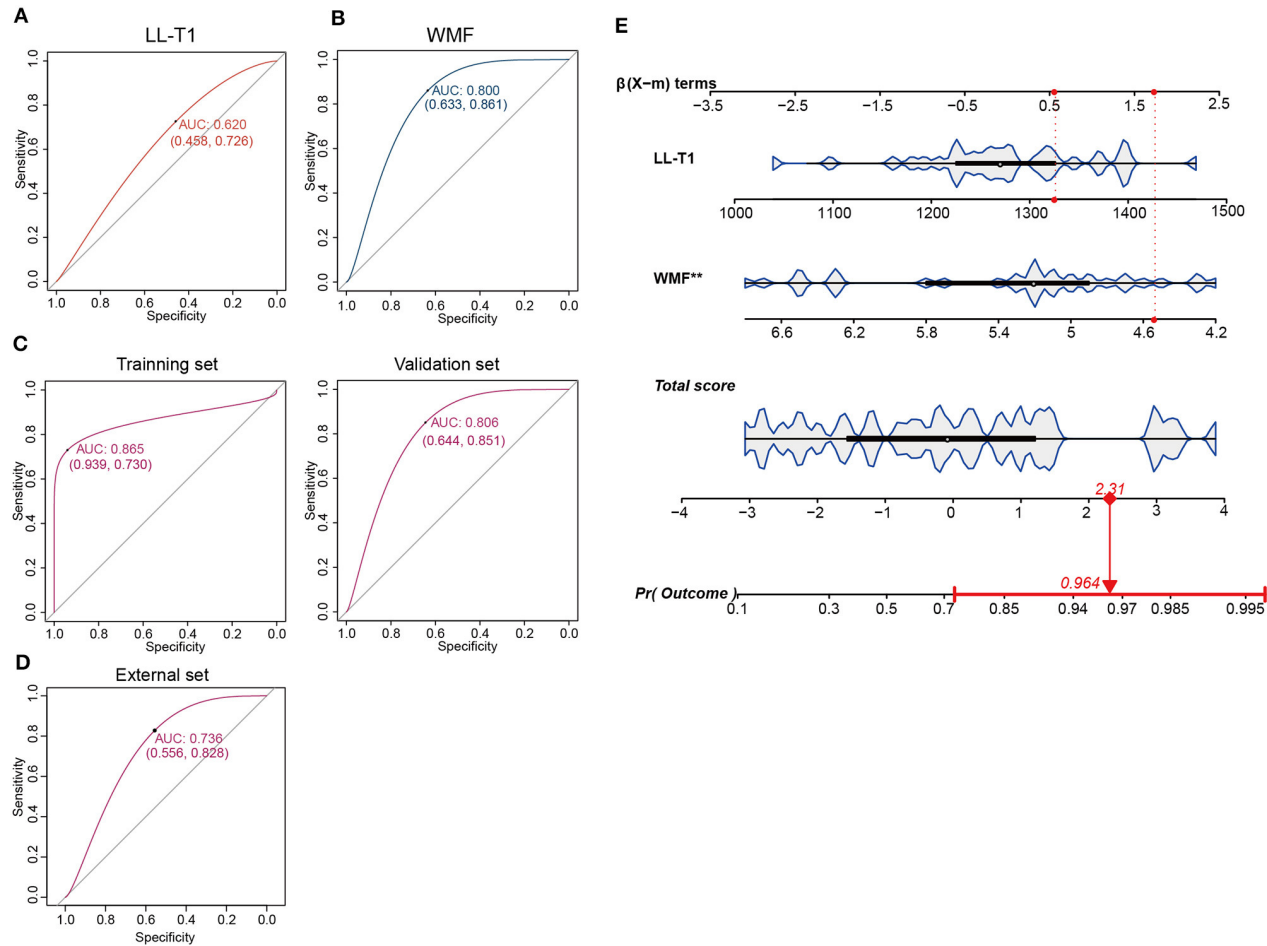
Construction and validation of the prediction model. **(A, B)** Area under the curve (AUC) for lateral lemniscus T1 value (LL-T1) and white matter fraction (WMF). **(C)** AUC for the training and validation sets. **(D)** AUC for the external set. **(E)** Nomogram illustrating the model for clinical application.

Based on the aforementioned findings, we identified seven parameters (IC-T1, LL-T1, MCP-T1, WMV, MYV, MYF, and WMF) correlated with SNHL. Subsequently, we evaluated the predictive value of these parameters for SNHL. To achieve this, we randomly divided 61 samples at 3-month group into training and validation sets. Through univariate and multivariate analysis, we identified two independent risk factors, LL-T1 and WMF (Table 9). We then assessed the predictive performance of LL-T1 and WMF, resulting in respective AUCs of 0.620 and 0.800, respectively (Figures 6A, B). Next, we combined LL-T1 and WMF to construct a model, yielding AUCs of 0.865 and 0.806 for the training and validation sets, respectively (Figure 6C), indicating favorable performance. To further access the performance of the model, we conducted an external set. The AUC for external set was 0.736 (Figure 6D). To facilitate clinical application, we developed a nomogram for visualizing the model, enabling doctors to calculate predicted scores based on LL-T1 and WMF values and thereby predict the probability of SNHL (Figure 6E).

**Table 9:** Univariate and multivariate analysis of parameters correlated with SNHL.

	**Univariate analysis**		**Multivariate analysis**	
**Variables**	**OR (95%CI)**	***P*** **-value**	**OR (95%CI)**	***P*** **-value**
WMV	0.63 (0.41–0.95)	0.029		
MYV	0.62 (0.36–1.05)	0.076		
WMF	0.17 (0.05–0.62)	0.007	0.12 (0.03–0.54)	0.006
MYF	0.02 (0.00–0.73)	0.033		
IC-T1	1.01 (1.00–1.01)	0.121		
LL-T1	1.01 (1.00–1.02)	0.129	1.01 (1.00–1.02)	0.070
MCP-T1	1.00 (1.00–1.01)	0.203		

WMV, volume of white matter; MYV, myelination volume; WMF, white matter fraction; MYF, myelin fraction; IC, inferior colliculus; LL, lateral lemniscus; MCP, middle cerebellar peduncle.

## Discussion

SNHL manifests before language acquisition, potentially impeding linguistic development. The absence of auditory stimuli from birth in SNHL children may disrupt language learning and alter the formation of neural pathways, leading to structural changes in the brain (Chari and Chan, 2017). Late detection of hearing impairment in infants and young children with SNHL can result in profound learning and developmental challenges. Some studies have shown that high risk factors that correlated with onset of SNHL, including preterm birth, low birth weight infants, hyperbilirubinemia, cytomegalovirus infection, inner ear abnormalities, etc. (Wroblewska-Seniuk et al., 2018; Alhazmi, 2023). Our research results indicated that NH and IEM are high-risk factors for SNHL, possibly due to our analysis being not performed in the general population but in one tertiary care hospital, where there is a big neurological intensive.

Previous investigations into macrostructural disparities between deaf individuals and those without hearing loss revealed diminished WMV but preserved gray matter volume in the auditory cortex, particularly in Heschl's gyrus (HG) and the adjacent temporal lobe. However, WMV exhibited inconsistent patterns across left and right brain hemispheres, with the most significant differences observed in the right posterior superior temporal gyrus (Hribar et al., 2014; Karns et al., 2017). Moreover, microstructural changes in white matter have been documented in individuals with hearing loss during various stages of life (Miao et al., 2013; Park et al., 2018; Kim et al., 2021). Yet, limited knowledge exists regarding white matter microstructural properties in children with SNHL. Research suggests that the gray matter volume of the right hemisphere, alongside white matter volume, is more susceptible to impairment compared to the left hemisphere (Manno et al., 2021). In our study, we found that quantitative T1 values were higher in children with SNHL than in their normally hearing counterparts. These discrepancies were observed in multiple brain regions, including the IC, LL, and MCP. Elevated quantitative T1 values signify alterations in myelin microstructure. Notably, our investigation is the first to report differences in T1 values among children with SNHL within the first 3 months of life. Analysis of whole brain volume revealed lower values of WMV, WMF, MYV, and MYF in children born with SNHL. Additionally, these parameters displayed a positive correlation with age. Compared to the control group, children with SNHL exhibited reduced WMV, WMF, MYV, and MYF.

Brain development follows a sequential pattern, with myelin sheath formation initiating around the fifth month of fetal development and progressing alongside central nervous system myelination, continuing throughout life (Mukherjee et al., 2001). White matter myelination typically commences between 6 and 8 months, with most white matter achieving maturity in myelin sheath formation by 18 months (Mukherjee et al., 2001; Barkovich, 2005; Lebel and Deoni, 2018). The observed increase in T1 values could be attributed to hearing impairment, which may impede the normal pace of development and maturation in these regions. Literature suggests that white matter development in infants follows a trajectory from dorsal to ventral, caudal to cephalic, and from central to peripheral regions (Shi et al., 2019). Early developmental activity is notable in areas such as the IC, LL, and MCP.

Assessing myelination plays a pivotal role in evaluating neurological development (Khelfaoui et al., 2024). SyMRI offers enhanced capabilities in detecting MS plaques compared to conventional MRI methods (Miller et al., 1998; Granberg et al., 2016; Hagiwara et al., 2017). Utilizing synthetic DIR and PSIR images can facilitate the identification of intra-cortical or mixed white matter-gray matter lesions (Miller et al., 1998). Vagberg et al. demonstrated the validity and reproducibility of SyMRI volumetric analysis in determining BPF in MS (Vagberg et al., 2013, 2016). In pediatric MS, BPF is notably lower compared to adult MS, primarily attributed to gray matter loss (Vagberg et al., 2013). Notably, our study found no significant differences in brain segmentation-related indices between the control and SNHL groups at 1.5 months, suggesting a relatively minor impact of SNHL on brain development at this early age. However, by 3 months, we observed no significant differences between the control and mild SNHL groups, indicating a minor effect of mild SNHL on brain development. In contrast, the moderate and severe SNHL groups exhibited significant reductions in WMV and myelination-related measures, indicating distinct structural alterations with increasing severity of SNHL. This corroborates previous research; Smith et al. (2011) observed decreased white matter in the anterior Heschl's gyrus in individuals with hearing loss using whole-brain voxel-based morphometry. Notably, our study focused on children as young as 3 months, utilizing SyMRI to detect subtle changes in brain development associated with SNHL, underscoring the impact of early hearing abnormalities on neurological development.

Kim et al. (2017) identified that the myelin volume percentage automatically generated by SyMRI within the brain substance volume closely adhered to the established myelin maturation Gompertz model and exhibited strong correlations with R1 and R2 relaxation rates. The quantification of myelin using SyMRI presents a promising avenue for assessing brain development in children. In a study utilizing VBM, Hribar et al. (2020) observed a significant decrease in WM volume within the left medial frontal gyrus and the right suboccipital gyrus in deaf patients, with no notable difference in gray matter volume, aligning with our findings. Notably, our study unveils differences in white matter occurring before language development, particularly in subjects around 3 months old with moderate-to-severe SNHL, a phenomenon not documented in existing literature. These findings suggest potential neuroplastic changes linked to brain reorganization following early hearing deprivation in SNHL infants.

A nomogram is a graphical tool which is commonly used to estimate prognosis in oncology and medicine. With the ability to generate an individual numerical probability of a clinical event by integrating diverse prognostic and determinant variables. Rapid computation through user friendly digital interfaces, together with increased accuracy, and more easily understood prognoses, allow for seamless incorporation of nomogram derived prognosis to aid in clinical decision making. This has led to the ubiquitous appearance of nomogram in clinical use (Ohori Tatsuo et al., 2009; Balachandran et al., 2015; Gandaglia et al., 2019). This study we constructed a prediction model based on two factors key SyMRI quantitative parameters LL-T1 and WMF, for distinguishing SNHL. This is easy for clinical doctors to calculate quantitative values of LL-T1 and WMF and arrange them horizontally on the column chart with scaled line segments in their respective proportions. By calculating the total score values corresponding to each parameter and finding the corresponding predicted risk values below the total score scale, we can quickly obtain the prediction probability for SNHL.

The molecular mechanisms through which sensorineural hearing loss (SNHL) impacts brain development are still not fully understood. Traditionally, it was believed that SNHL primarily targets hair cells, with cochlear nerve loss considered secondary to hair cell degeneration. However, in cases of noise-induced hearing loss, even reversible threshold shifts (without hair cell loss) can result in permanent loss of over 50% of cochlear nerve/hair cell synapses. Similarly, in age-related hearing loss, the degeneration of cochlear synapses precedes both hair cell loss and threshold elevation (Kujawa and Liberman, 2015). There are reports indicating the possibility of spontaneous re-innervation (Puel, 1995; Pujol and Puel, 1999; Sun et al., 2001), or that some immediate synapse loss may be reversible (Liu et al., 2012; Shi et al., 2013, 2015, 2016). However, how these molecular changes manifest in SyMRI imaging remains unclear. Ongoing research aims to delve deeper into this phenomenon, elucidate its underlying mechanisms, and evaluate the potential effectiveness of therapeutic interventions.

This study is subject to certain limitations. Firstly, the sample size was relatively small, potentially impacting the statistical power and the generalizability of the research findings. Secondly, there was no follow-up conducted to assess the long-term intellectual and behavioral development of the SNHL patient group. Long-term follow-up could shed light on the impact of SNHL on various aspects such as language proficiency, motor skills, and learning abilities across different age groups, highlighting the necessity for further investigation. Thirdly, due to our hospital being a provincial key maternal and child health hospital, there may be bias in sample selection.

## Conclusion

In conclusion, T1 values, coupled with measurements of WMV, MYV, WMF, and MYF, hold promise as potential indicators for early detection of brain development anomalies in children with SNHL. Quantitative assessments in areas such as the IC, LL, and MCP could assist in distinguishing patients with moderate to severe SNHL. Moreover, observed reductions in WMV and myelin levels may serve as predictive factors for the progression of moderate and severe SNHL in pediatric populations.

## Data availability statement

The original contributions presented in the study are included in the article/Supplementary material, further inquiries can be directed to the corresponding author.

## Ethics statement

The studies involving humans were approved by The Third Affiliated Hospital of Zhengzhou University. The studies were conducted in accordance with the local legislation and institutional requirements. Written informed consent for participation in this study was provided by the participants' legal guardians/next of kin.

## Author contributions

PZ: Investigation, Writing – original draft, Writing – review & editing, Data curation, Formal analysis, Methodology, Project administration, Resources, Software, Validation, Visualization. JY: Data curation, Formal analysis, Methodology, Project administration, Software, Writing – original draft. YS: Data curation, Formal analysis, Investigation, Methodology, Software, Writing – original draft. MC: Formal analysis, Methodology, Software, Writing – review & editing. XZhao: Formal analysis, Methodology, Writing – review & editing. KW: Methodology, Writing – review & editing, Data curation. LL: Methodology, Writing – review & editing, Software. QX: Software, Writing – review & editing. GN: Writing – review & editing, Data curation. LM: Writing – review & editing, Methodology. XW: Methodology, Writing – review & editing. LZ: Writing – review & editing, Formal analysis. XZhan: Writing – review & editing, Conceptualization, Funding acquisition, Investigation, Supervision, Writing – original draft.

## Supplementary material

Supplementary Figure 1 Comparison of T1 values in the inferior colliculus (IC), lateral lemniscus (LL), and middle cerebellar peduncle (MCP) among four groups of children at 1.5 and 3 months. **(A–C)** Comparison of T1 values among Normal-NH, Normal-Non-NH, SNHL-NH, and SNHL-Non-NH, subgroups at 1.5 months. **(D–F)** Comparison of T1 values among Normal-NH, Normal-Non-NH, SNHL-NH, and SNHL-Non-NH, subgroups at 3 months. T1 values are presented in milliseconds.

Supplementary Figure 2 Comparison of white matter volume (WMV), white matter fraction (WMF), myelin volume (MYV), and myelin fraction (MYF) among Normal-NH, Normal-Non-NH, SNHL-NH, and SNHL-Non-NH, subgroups at 1.5 and 3 months. **(A–D)** Comparison at 1.5 months. **(E–H)** Comparison at 3 months.

## References

[B1] Alhazmi W. (2023). Risk factors associated with hearing impairment in infants and children: a systematic review. Cureus 15:e40464. 10.7759/cureus.40464 37456446 PMC10349545

[B2] Andica C. Hagiwara A. Nakazawa M. Kumamaru K. K. Hori M. Ikeno M.. (2017). Synthetic MR imaging in the diagnosis of bacterial meningitis. Magn. Reson. Med. Sci. 16, 91–92. 10.2463/mrms.ci.2016-0082 28003620 PMC5600066

[B3] Andica C. Hagiwara A. Nakazawa M. Tsuruta K. Takano N. Hori M.. (2016). The advantage of synthetic MRI for the visualization of early white matter change in an infant with Sturge-Weber Syndrome. Magn. Reson. Med. Sci. 15, 347–348. 10.2463/mrms.ci.2015-0164 27001386 PMC5608107

[B4] Badve C. Yu A. Dastmalchian S. Rogers M. Ma D. Jiang Y.. (2017). MR fingerprinting of adult brain tumors: initial experience. Am. J. Neuroradiol. 38, 492–499. 10.3174/ajnr.A5035 28034994 PMC5352493

[B5] Balachandran V. P. Gonen M. Smith J. J. Dematteo R. P. (2015). Nomograms in oncology: more than meets the eye. Lancet Oncol. 16, e173–e180. 10.1016/S1470-2045(14)71116-7 25846097 PMC4465353

[B6] Barkovich A. J. (2005). Magnetic resonance techniques in the assessment of myelin and myelination. J. Inherit. Metab. Dis. 28, 311–343. 10.1007/s10545-005-5952-z 15868466

[B7] Chari D. A. Chan D. K. (2017). Diagnosis and treatment of congenital sensorineural hearing loss. Curr. Otorhinolaryngol. Rep. 5, 251–258. 10.1007/s40136-017-0163-3 29761033 PMC5947965

[B8] Chen Y. Su S. Dai Y. Wen Z. Qian L. Zhang H.. (2021). Brain volumetric measurements in children with attention deficit hyperactivity disorder: a comparative study between synthetic and conventional magnetic resonance imaging. Front. Neurosci. 15:711528. 10.3389/fnins.2021.711528 34759789 PMC8573371

[B9] Gandaglia G. Ploussard G. Valerio M. Mattei A. Fiori C. Fossati N.. (2019). A novel nomogram to identify candidates for extended pelvic lymph node dissection among patients with clinically localized prostate cancer diagnosed with magnetic resonance imaging-targeted and systematic biopsies. Eur. Urol. 75, 506–514. 10.1016/j.eururo.2018.10.012 30342844

[B10] Goncalves F. G. Serai S. D. Zuccoli G. (2018). Synthetic brain MRI: review of current concepts and future directions. Top. Magn. Reson. Imaging 27, 387–393. 10.1097/RMR.0000000000000189 30516691

[B11] Granberg T. Uppman M. Hashim F. Cananau C. Nordin L. E. Shams S.. (2016). Clinical feasibility of synthetic MRI in multiple sclerosis: a diagnostic and volumetric validation study. Am. J. Neuroradiol. 37, 1023–1029. 10.3174/ajnr.A4665 26797137 PMC7963550

[B12] Gulani V. Schmitt P. Griswold M. A. Webb A. G. Jakob P. M. (2004). Towards a single-sequence neurologic magnetic resonance imaging examination: multiple-contrast images from an IR TrueFISP experiment. Invest. Radiol. 39, 767–774. 10.1097/00004424-200412000-00008 15550838

[B13] Hagiwara A. Hori M. Yokoyama K. Takemura M. Y. Andica C. Tabata T.. (2017). Synthetic MRI in the detection of multiple sclerosis plaques. Am. J. Neuroradiol. 38, 257–263. 10.3174/ajnr.A5012 27932506 PMC7963841

[B14] Hribar M. Suput D. Battelino S. Vovk A. (2020). Review article: structural brain alterations in prelingually deaf. Neuroimage 220:117042. 10.1016/j.neuroimage.2020.117042 32534128

[B15] Hribar M. Suput D. Carvalho A. A. Battelino S. Vovk A. (2014). Structural alterations of brain grey and white matter in early deaf adults. Hear. Res. 318, 1–10. 10.1016/j.heares.2014.09.008 25262621

[B16] Ji S. Yang D. Lee J. Choi S. H. Kim H. Kang K. M. (2022). Synthetic MRI: technologies and applications in neuroradiology. J. Magn. Reson. Imaging 55, 1013–1025. 10.1002/jmri.27440 33188560

[B17] Johnson J. C. S. Marshall C. R. Weil R. S. Bamiou D. E. Hardy C. J. D. Warren J. D. (2021). Hearing and dementia: from ears to brain. Brain 144, 391–401. 10.1093/brain/awaa429 33351095 PMC7940169

[B18] Karns C. M. Stevens C. Dow M. W. Schorr E. M. Nevil,L E H. J. (2017). Atypical white-matter microstructure in congenitally deaf adults: a region of interest and tractography study using diffusion-tensor imaging. Hear. Res. 343, 72–82. 10.1016/j.heares.2016.07.008 27473505 PMC11668359

[B19] Khelfaoui H. Ibaceta-Gonzalez C. Angulo M. C. (2024). Functional myelin in cognition and neurodevelopmental disorders. Cell. Mol. Life Sci. 81:181. 10.1007/s00018-024-05222-2 38615095 PMC11016012

[B20] Kim E. Kang H. Han K. H. Lee H. J. Suh M. W. Song J. J.. (2021). Reorganized brain white matter in early- and late-onset deafness with diffusion tensor imaging. Ear Hear. 42, 223–234. 10.1097/AUD.0000000000000917 32833702

[B21] Kim H. G. Moon W. J. Han J. Choi J. W. (2017). Quantification of myelin in children using multiparametric quantitative MRI: a pilot study. Neuroradiology 59, 1043–1051. 10.1007/s00234-017-1889-9 28765995

[B22] Korver A. M. Konings S. Dekker F. W. Beers M. Wever C. C. Frijns J. H.. (2010). Newborn hearing screening vs later hearing screening and developmental outcomes in children with permanent childhood hearing impairment. JAMA 304, 1701–1708. 10.1001/jama.2010.1501 20959580

[B23] Kujawa S. G. Liberman M. C. (2015). Synaptopathy in the noise-exposed and aging cochlea: primary neural degeneration in acquired sensorineural hearing loss. Hear. Res. 330, 191–199. 10.1016/j.heares.2015.02.009 25769437 PMC4567542

[B24] Lebel C. Deoni S. (2018). The development of brain white matter microstructure. Neuroimage 182, 207–218. 10.1016/j.neuroimage.2017.12.097 29305910 PMC6030512

[B25] Liu L. Wang H. Shi L. Almuklass A. He T. Aiken S.. (2012). Silent damage of noise on cochlear afferent innervation in guinea pigs and the impact on temporal processing. PLoS ONE 7:e49550. 10.1371/journal.pone.0049550 23185359 PMC3504112

[B26] Manno F. A. M. Rodriguez-Cruces R. Kumar R. Ratnanather J. T. Lau C. (2021). Hearing loss impacts gray and white matter across the lifespan: systematic review, meta-analysis and meta-regression. Neuroimage 231:117826. 10.1016/j.neuroimage.2021.117826 33549753 PMC8236095

[B27] Miao W. Li J. Tang M. Xian J. Li W. Liu Z.. (2013). Altered white matter integrity in adolescents with prelingual deafness: a high-resolution tract-based spatial statistics imaging study. Am. J. Neuroradiol. 34, 1264–1270. 10.3174/ajnr.A3370 23275596 PMC7964594

[B28] Miller D. H. Grossman R. I. Reingold S. C. Mcfarland H. F. (1998). The role of magnetic resonance techniques in understanding and managing multiple sclerosis. Brain 121 ( Pt 1), 3–24. 10.1093/brain/121.1.3 9549485

[B29] Mukherjee P. Miller J. H. Shimony J. S. Conturo T. E. Lee B. C. Almli C. R.. (2001). Normal brain maturation during childhood: developmental trends characterized with diffusion-tensor MR imaging. Radiology 221, 349–358. 10.1148/radiol.2212001702 11687675

[B30] Nunez-Gonzalez L. Van Garderen K. A. Smits M. Jaspers J. Romero A. M. Poot D. H. J.. (2022). Pre-contrast MAGiC in treated gliomas: a pilot study of quantitative MRI. Sci. Rep. 12:21820. 10.1038/s41598-022-24276-5 36528673 PMC9759533

[B31] Ohori Tatsuo G. Riu Hamada M. Gondo T. Hamada R. (2009). Nomogram as predictive model in clinical practice. Gan To Kagaku Ryoho 36, 901–906. 19542708

[B32] Park K. H. Chung W. H. Kwon H. Lee J. M. (2018). Evaluation of cerebral white matter in prelingually deaf children using diffusion tensor imaging. Biomed Res. Int. 2018:6795397. 10.1155/2018/6795397 29511689 PMC5817214

[B33] Puel J. L. (1995). Chemical synaptic transmission in the cochlea. Prog. Neurobiol. 47, 449–476. 10.1016/0301-0082(95)00028-3 8787031

[B34] Pujol R. Puel J. L. (1999). Excitotoxicity, synaptic repair, and functional recovery in the mammalian cochlea: a review of recent findings. Ann. N. Y. Acad. Sci. 884, 249–254. 10.1111/j.1749-6632.1999.tb08646.x 10842598

[B35] Shende S. A. Mudar R. A. (2023). Cognitive control in age-related hearing loss: a narrative review. Hear. Res. 436:108814. 10.1016/j.heares.2023.108814 37315494

[B36] Shi J. Yang S. Wang J. Huang S. Yao Y. Zhang S.. (2019). Detecting normal pediatric brain development with diffusional kurtosis imaging. Eur. J. Radiol. 120:108690. 10.1016/j.ejrad.2019.108690 31605964

[B37] Shi L. Chang Y. Li X. Aiken S. J. Liu L. Wang J. (2016). Coding deficits in noise-induced hidden hearing loss may stem from incomplete repair of ribbon synapses in the cochlea. Front. Neurosci. 10:231. 10.3389/fnins.2016.00231 27252621 PMC4879136

[B38] Shi L. Liu K. Wang H. Zhang Y. Hong Z. Wang M.. (2015). Noise induced reversible changes of cochlear ribbon synapses contribute to temporary hearing loss in mice. Acta Otolaryngol. 135, 1093–1102. 10.3109/00016489.2015.1061699 26139555

[B39] Shi L. Liu L. He T. Guo X. Yu Z. Yin S.. (2013). Ribbon synapse plasticity in the cochleae of Guinea pigs after noise-induced silent damage. PLoS ONE 8:e81566. 10.1371/journal.pone.0081566 24349090 PMC3857186

[B40] Slade K. Plack C. J. Nuttall H. E. (2020). The effects of age-related hearing loss on the brain and cognitive function. Trends Neurosci. 43, 810–821. 10.1016/j.tins.2020.07.005 32826080

[B41] Smith K. M. Mecoli M. D. Altaye M. Komlos M. Maitra R. Eaton K. P.. (2011). Morphometric differences in the Heschl's gyrus of hearing impaired and normal hearing infants. Cereb. Cortex 21, 991–998. 10.1093/cercor/bhq164 20841321 PMC3114550

[B42] Sun H. Hashino E. Ding D. L. Salvi R. J. (2001). Reversible and irreversible damage to cochlear afferent neurons by kainic acid excitotoxicity. J. Comp. Neurol. 430, 172–181. 10.1002/1096-9861(20010205)430:2<172::AID-CNE1023>3.0.CO;2-W 11135254

[B43] Surprenant A. M. Didonato R. (2014). Community-dwelling older adults with hearing loss experience greater decline in cognitive function over time than those with normal hearing. Evid. Based Nurs. 17, 60–61. 10.1136/eb-2013-101375 23842726

[B44] Vagberg M. Ambarki K. Lindqvist T. Birgander R. Svenningsson A. (2016). Brain parenchymal fraction in an age-stratified healthy population - determined by MRI using manual segmentation and three automated segmentation methods. J. Neuroradiol. 43, 384–391. 10.1016/j.neurad.2016.08.002 27720265

[B45] Vagberg M. Lindqvist T. Ambarki K. Warntjes J. B. Sundstrom P. Birgander R.. (2013). Automated determination of brain parenchymal fraction in multiple sclerosis. Am. J. Neuroradiol. 34, 498–504. 10.3174/ajnr.A3262 22976234 PMC7964911

[B46] Van Der Weijden C. W. J. Biondetti E. Gutmann I. W. Dijkstra H. Mckerchar R. De Paula Faria D.. (2023). Quantitative myelin imaging with MRI and PET: an overview of techniques and their validation status. Brain 146, 1243–1266. 10.1093/brain/awac436 36408715 PMC10115240

[B47] Vanderhasselt T. Naeyaert M. Watte N. Allemeersch G. J. Raeymaeckers S. Dudink J.. (2020). Synthetic MRI of preterm infants at term-equivalent age: evaluation of diagnostic image quality and automated brain volume segmentation. Am. J. Neuroradiol. 41, 882–888. 10.3174/ajnr.A6533 32299803 PMC7228170

[B48] Wang H. Liang Y. Fan W. Zhou X. Huang M. Shi G.. (2019). DTI study on rehabilitation of the congenital deafness auditory pathway and speech center by cochlear implantation. Eur. Arch. Otorhinolaryngol. 276, 2411–2417. 10.1007/s00405-019-05477-7 31127414 PMC6682568

[B49] Wang Y. Xiong W. Sun X. Lu K. Duan F. Wang H.. (2023). Impact of environmental noise exposure as an inducing factor on the prognosis of sudden sensorineural hearing loss: a retrospective case-control study. Front. Neurosci. 17:1210291. 10.3389/fnins.2023.1210291 37457012 PMC10339706

[B50] Warntjes J. B. Leinhard O. D. West J. Lundberg P. (2008). Rapid magnetic resonance quantification on the brain: optimization for clinical usage. Magn. Reson. Med. 60, 320–329. 10.1002/mrm.21635 18666127

[B51] West J. Warntjes J. B. Lundberg P. (2012). Novel whole brain segmentation and volume estimation using quantitative MRI. Eur. Radiol. 22, 998–1007. 10.1007/s00330-011-2336-7 22113264

[B52] Wroblewska-Seniuk K. Dabrowski P. Greczka G. Szabatowska K. Glowacka A. Szyfter W.. (2018). Sensorineural and conductive hearing loss in infants diagnosed in the program of universal newborn hearing screening. Int. J. Pediatr. Otorhinolaryngol. 105, 181–186. 10.1016/j.ijporl.2017.12.007 29447811

[B53] Yeh E. A. Weinstock-Guttman B. Ramanathan M. Ramasamy D. P. Willis L. Cox J. L.. (2009). Magnetic resonance imaging characteristics of children and adults with paediatric-onset multiple sclerosis. Brain 132, 3392–3400. 10.1093/brain/awp278 19892770

