# Supplementary figures and images for: The value of synthetic MRI in detecting the brain changes and hearing impairment of children with sensorineural hearing loss
Source: Front Neurosci. 2024 Jun 11;18:1365141. doi: 10.3389/fnins.2024.1365141 (PMC11197400; doi:10.3389/fnins.2024.1365141)

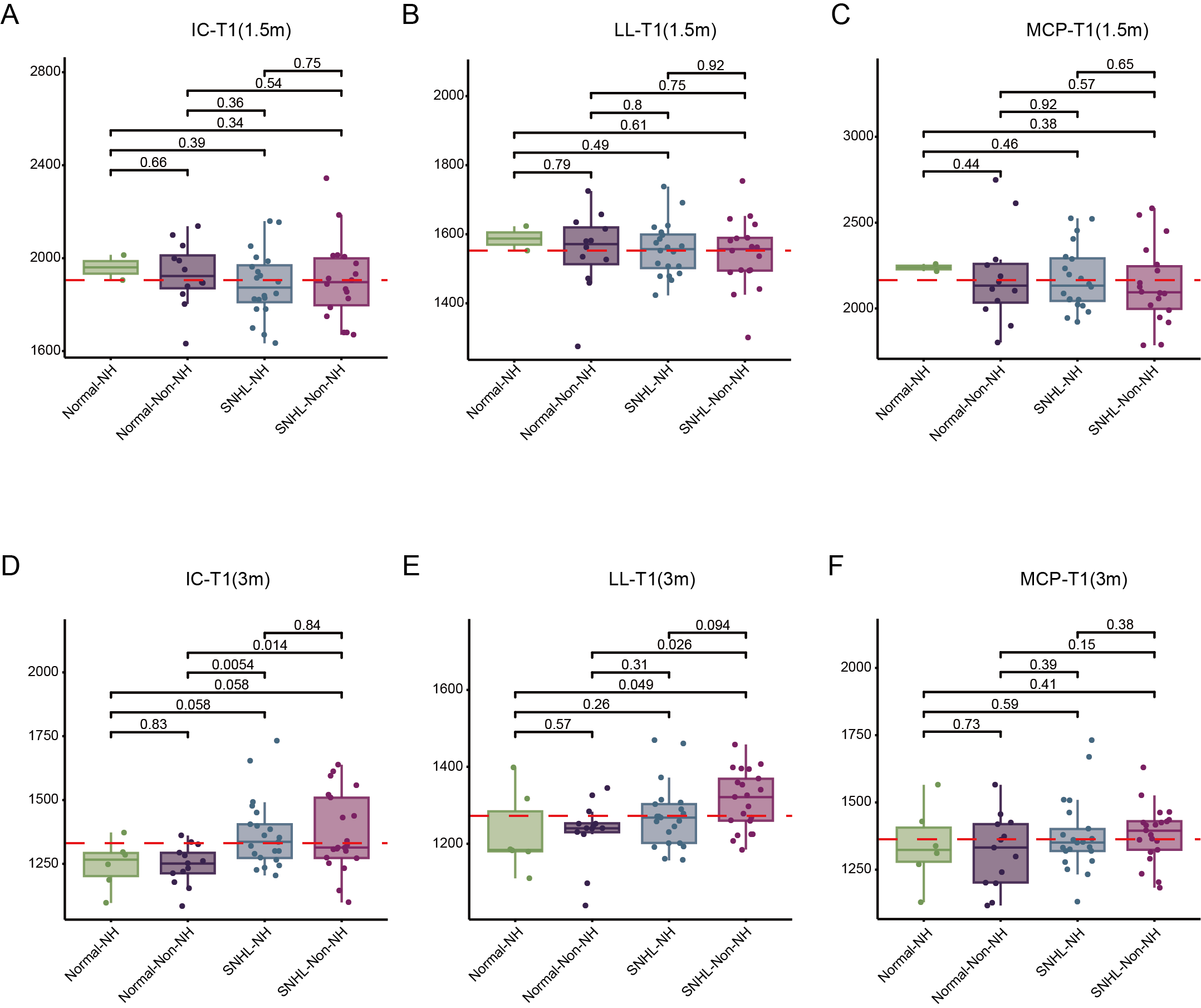

Supplement: Supplementary Figure 1 — Comparison of T1 values in the inferior colliculus (IC), lateral lemniscus (LL), and middle cerebellar peduncle (MCP) among four groups of children at 1.5 and 3 months. (A–C) Comparison of T1 values among Normal-NH, Normal-Non-NH, SNHL-NH, and SNHL-Non-NH, subgroups at 1.5 months. (D–F) Comparison of T1 values among Normal-NH, Normal-Non-NH, SNHL-NH, and SNHL-Non-NH, subgroups at 3 months. T1 values are presented in milliseconds. [file Image_1.TIF]

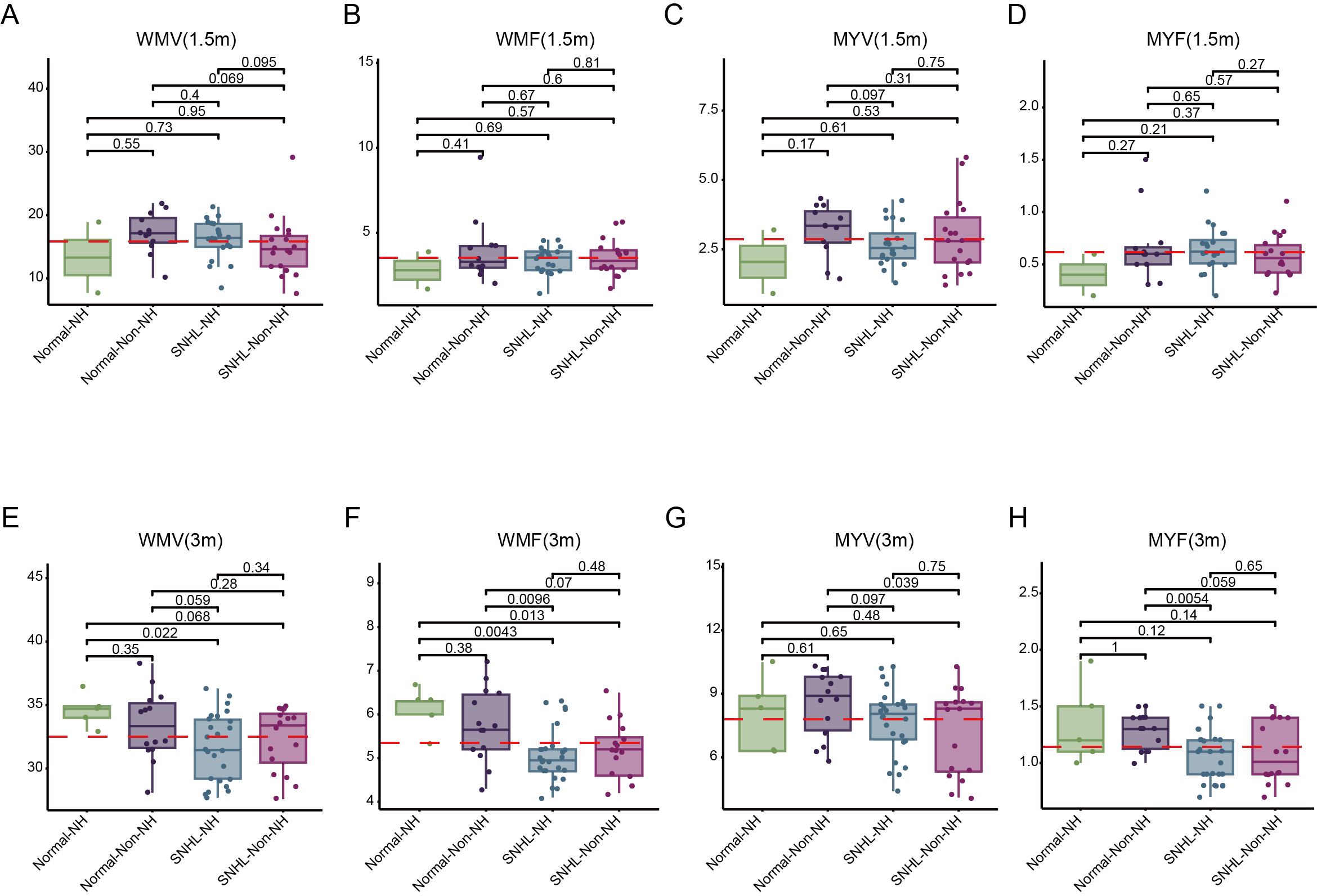

Supplement: Supplementary Figure 2 — Comparison of white matter volume (WMV), white matter fraction (WMF), myelin volume (MYV), and myelin fraction (MYF) among Normal-NH, Normal-Non-NH, SNHL-NH, and SNHL-Non-NH, subgroups at 1.5 and 3 months. (A–D) Comparison at 1.5 months. (E–H) Comparison at 3 months. [file Image_2.TIF]
